# From obscurity to prominence in the scope world

**DOI:** 10.1016/j.igie.2024.10.006

**Published:** 2024-10-22

**Authors:** Taisuke Fujita, Linda S. Lee

**Affiliations:** 1 Fujifilm Healthcare Americas Corporation, Ohio, United States; 2 Division of Gastroenterology, Hepatology and Endoscopy, Brigham and Women’s Hospital, 75 Francis Street, Boston, MA 02115

## Editor’s Introduction

Gastroenterologists take the endoscope for granted, although it is an incredibly complex instrument incorporating a light source with video optics and flexible tubing to allow complex movements and passage of equipment. The first endoscope dates back to 1853 by Desormeaux and was used to explore the urethra and bladder.^1^ Then, in 1868 Kussmaul developed the first gastroscopy.^2^ Countless iterations and innovations occurred in between, most notably with the advent of fiberoptic endoscopes by Larry Curtiss and Basil Hirschowitz in 1958 and of course the charged coupled device enabling projection of images onto a television screen, and we have our familiar endoscopes that form the backbone of what we offer as gastroenterologists.^2^ A handful of companies make these complex instruments. In the United States, reusable endoscope companies mainly include Olympus (Tokyo, Japan) and Pentax Medical (Tokyo, Japan), but recently Fujfilm (Tokyo, Japan) has made strong inroads with its array of endoscopes. I am delighted to speak with Tai Fujita, General Manager of Endoscopy and Vice President for Fujifilm Healthcare Americas Corporation.

Since 2018, Taisuke (Tai) Fujita has led the business strategy, market development, sales, and results performance for Fujifilm’s endoscopy division in the United States, driving year over year double-digital growth and increasing market share in hospital systems, academic medical systems, and ambulatory surgical centers across the country. With more than 20 years of experience with Fujifilm and its technologies, Tai’s focus is to continually foster endoscopic practice advancements in the United States through innovative and differentiated endoscopic technologies and by leading cross-functional teams to consistently deliver industry-proven service and support for Fujifilm customers.

Section Editor: Linda S. Lee, MD

**Linda Lee (L.L.): Thank you very much for making time with your incredibly busy schedule to discuss the history of Fujifilm Healthcare in endoscopy. Although Fujifilm endoscopes have been around for decades, they have not had much of a presence in the United States. Would you review the history of endoscopic technology within Fujifilm Healthcare?**

**Figure 1:**
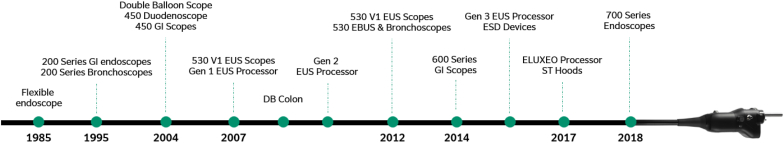
Fujifilm Endoscopy timeline of technology introductions to the U.S. market, 1985 to 2018. *DB*, Double balloon; *EBUS*, endobronchial US; *ESD*, endoscopic submucosal dissection; *ST*, short tapered.

**Figure 2:**
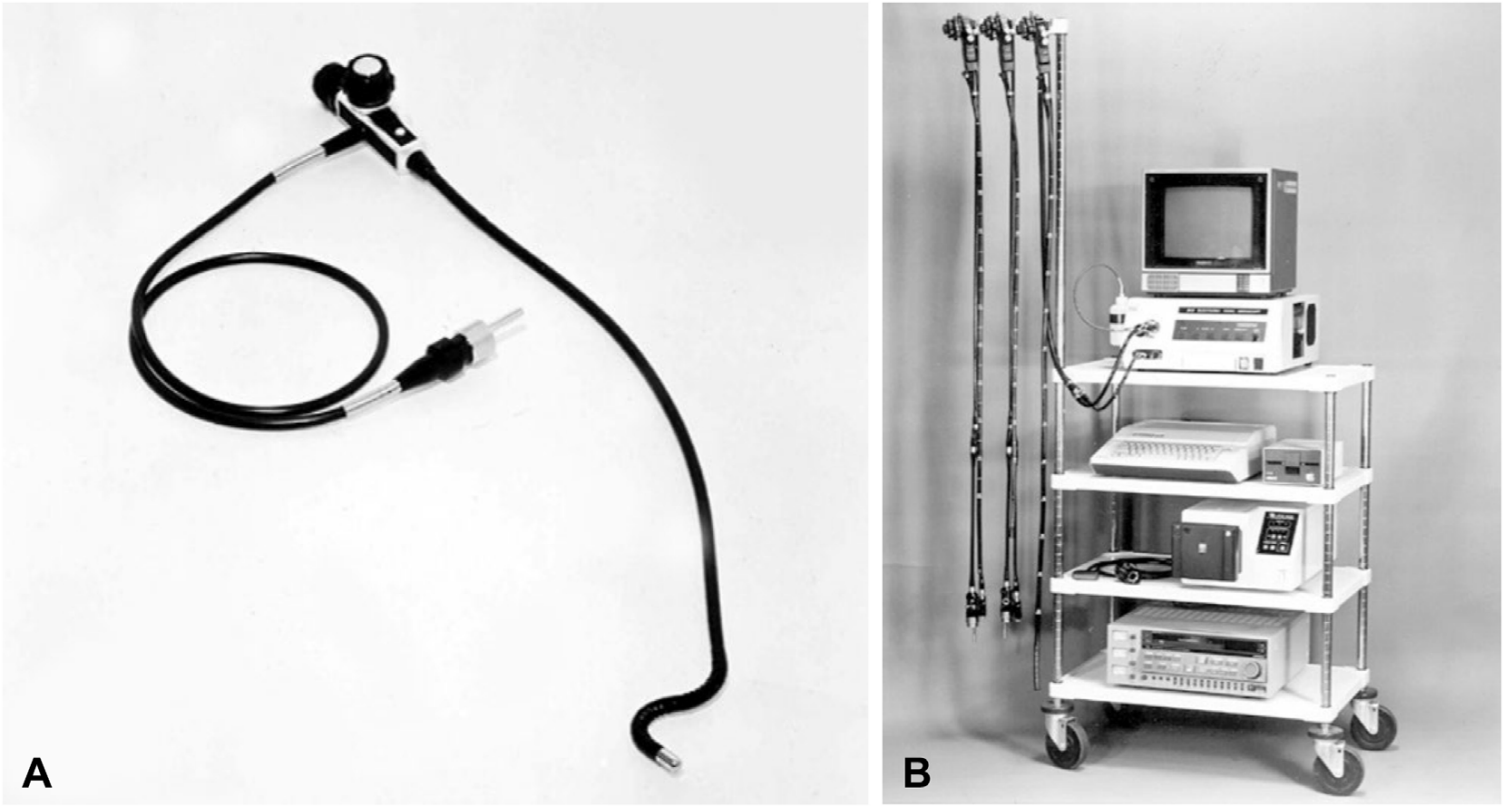
**A,** Fujifilm’s first fiberoptic endoscope in 1971. **B,** Fujifilm’s first video endoscope in 1984.

**Tai Fujita (T.F.):** Fujifilm introduced its first endoscope in the 1980s, and in the 1980s and 1990s our presence in U.S. endoscopy units in hospitals and ambulatory surgical centers was very limited (Figs. 1 and 2). The primary reason was that Fujifilm lacked a broad portfolio of endoscopes in the U.S. market to address routine and complex procedures. So too, the ergonomics of our endoscope felt different from other endoscope manufacturers. Different isn’t necessarily bad, but if it’s different from the endoscope you trained on in fellowship or have been using in your practice, that difference may be difficult to embrace and overcome.

**L.L.: Tell me about lessons learned from what happened with the virtual absence of Fujifilm endoscopes from the U.S. market until recently?**

**Figure 3:**
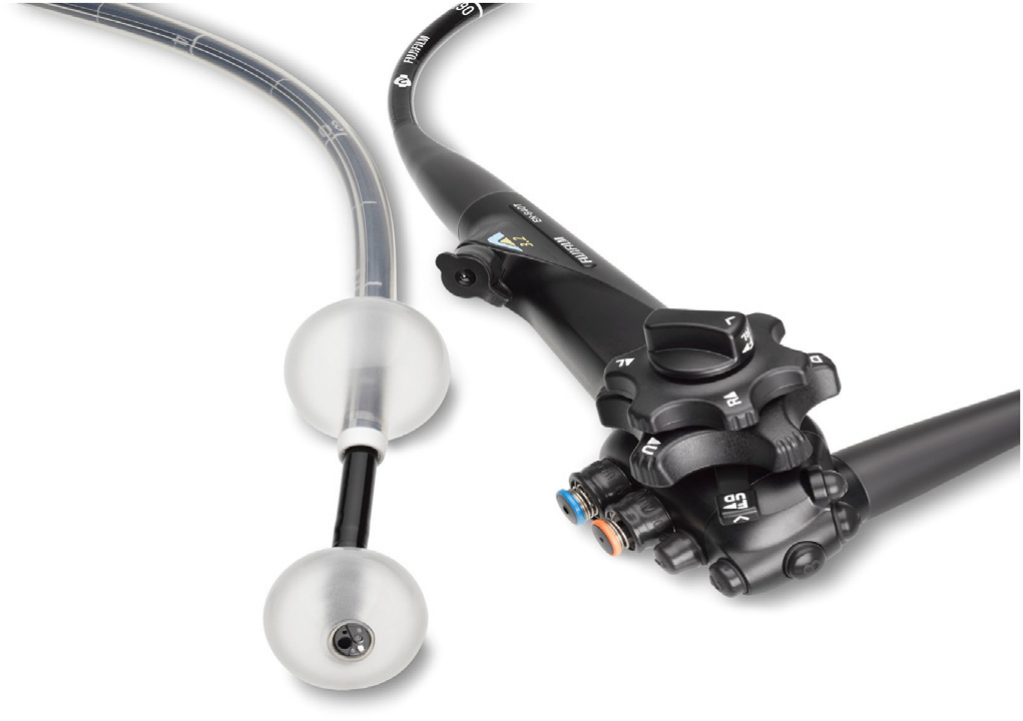
Fujifilm double-balloon endoscope.

**T.F.:** Over time, Fujifilm did introduce new endoscopes to the U.S. market, starting with 200 Series scope, and additional iterations were introduced over the next 30 years. Although Fujifilm has consistently been known for providing a bright and detailed endoscopic image, the types of scopes that Fujifilm offered was limited. Not intentionally, Fujifilm became known as the provider of double-balloon enteroscopy, a novel and niche scope that has become the criterion standard for endoscopists to provide noninvasive small-bowel diagnosis and treatment (Fig. 3). Numerous hospitals purchased this innovative scope but would not consider converting their gastroenterology suites given the incomplete portfolio of scopes and differing scope ergonomics.

**Figure 4:**
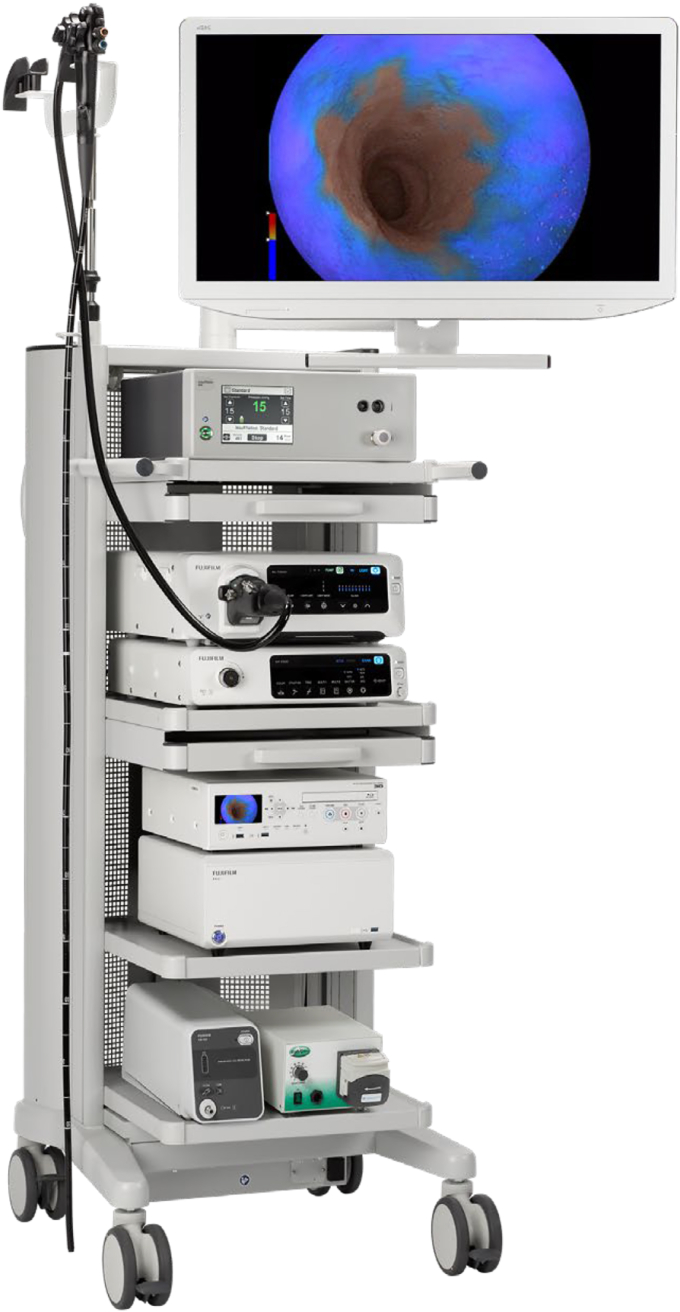
Fujifilm ELUXEO Vision Tower with scope.

All that started to change in 2018 with Fujifilm’s introduction of the ELUXEO Endoscopic Imaging System (Fig. 4) and its line of 700 Series colonoscopes and gastroscopes to the U.S. market. Fujifilm realized it needed to compete in a different way. Fujifilm leadership understood that to succeed we needed to differentiate on our founding principles of innovation, so we reimagined what innovation could mean to the gastroenterology physician. Endoscopic imaging technology from other manufacturers had not evolved in nearly a decade at that time. Fujifilm ELUXEO came to market offering the first of its kind LED multilight technology as well as impressive multizoom optics, enhanced imaging light modes that were clinically proven to improve adenoma detection and lesion characterization, along with new scope features that enhanced control, access, maneuverability, and ergonomics. That unique combination of benefits started to change the conversations we were having with gastroenterologists and the way they could now think about visualization through image clarity and system capabilities to help improve practice performance.

**L.L.: During this time, would you talk about what was happening with Fujifilm Healthcare and endoscopy globally outside of the United States?**

**Figure 5:**
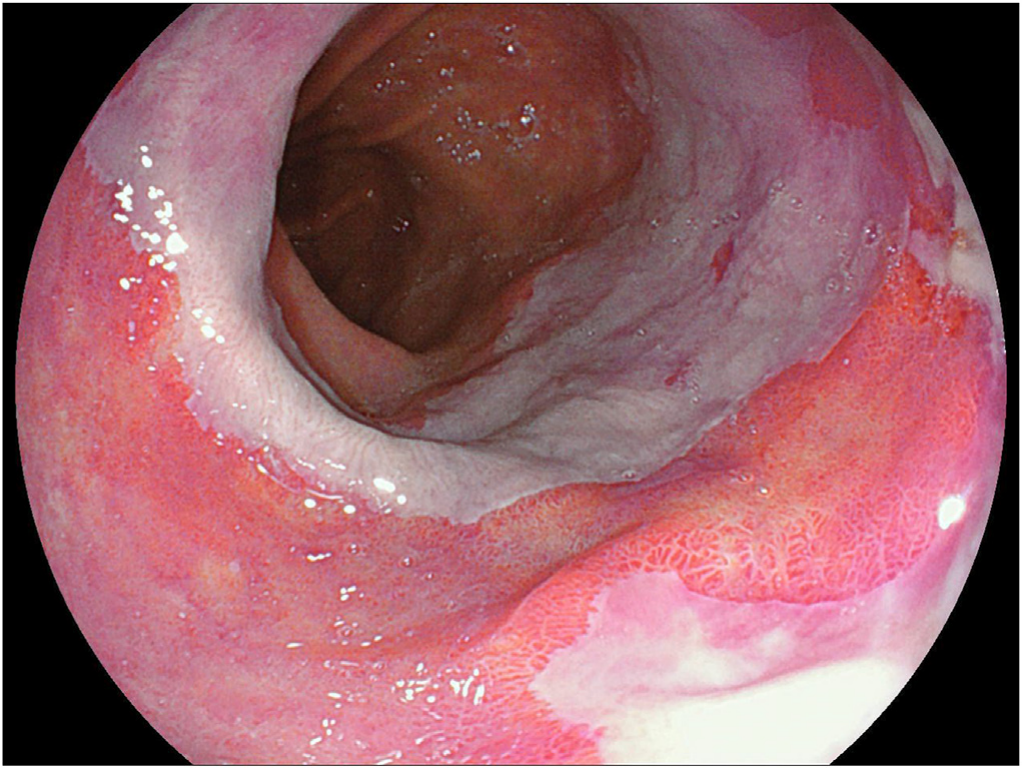
Barrett’s esophagus as seen using the Fujifilm linked-color imaging ELUXEO.

**Figure 6:**
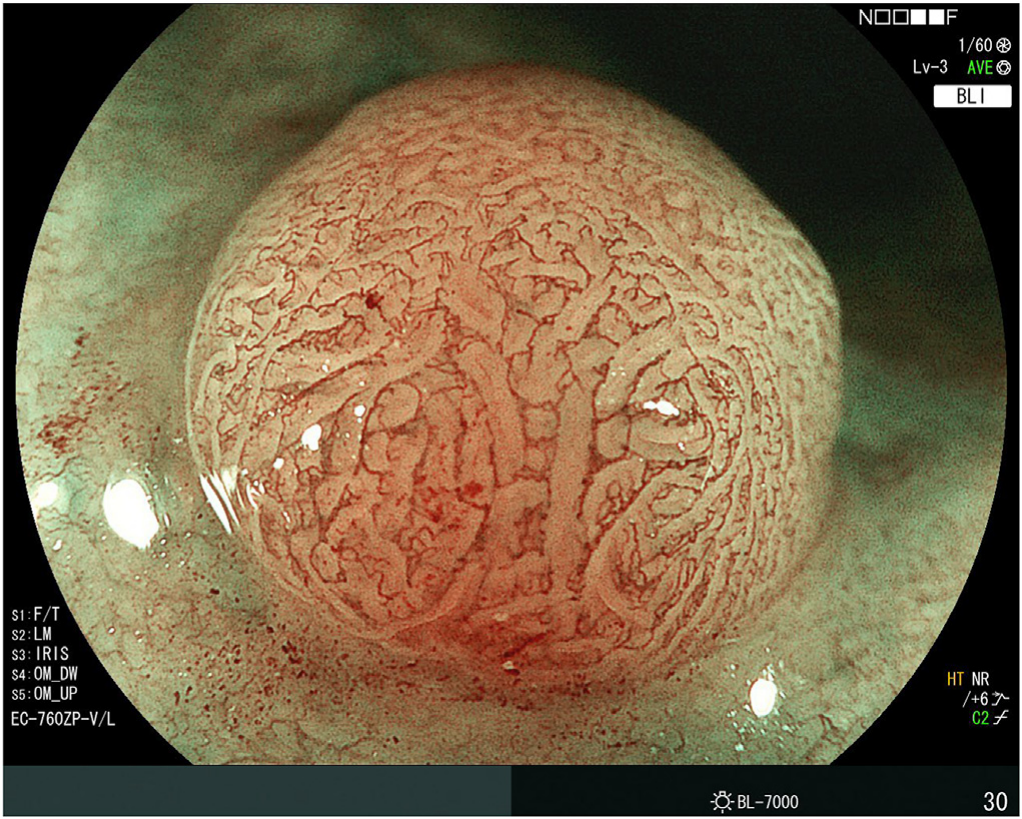
Fujifilm zoom endoscope image.

**T.F.:** Outside of the United States, Fujifilm saw a resurgence in clinicians adopting our endoscopic technology, beginning in 2017 when the ELUXEO Endoscopic Imaging System was first introduced to the global market. Because Asian and European markets were the first to experience the visible difference that the new ELUXEO system could deliver, a number of new differentiated innovations were rapidly being introduced. ELUXEO’s LED multilight technology with linked-color imaging and blue-light imaging prompted many clinical studies to be conducted and published—to date more than 100 across the globe—on the advantages of using linked-color imaging for adenoma detection and blue-light imaging for characterization and margin delineation (Fig. 5). Fujifilm’s optical multizoom endoscopes enabling high-quality optical zoom capabilities were also gaining attention and use in Asian and European markets as they provided optical magnification up to 145 times on a 26-inch monitor—more than 2 times what was previously offered (Fig. 6).

**L.L.: I’m interested to hear about the timing and reasoning behind the decision to invest in the U.S. market more recently.**

**T.F.:** With the success of the Fujifilm ELUXEO Endoscopic Imaging System taking hold in Asian and European markets, Fujifilm knew that the U.S. market would also be ripe for introducing endoscopic innovation. We affirmed this from speaking to many prominent U.S. gastroenterologists and advanced endoscopists who were eager for a change, and to be able to work with industry to develop and use new endoscopic tools that could help them explore new techniques for care delivery in increasingly complex procedures, like third-space endoscopy and endobariatrics. They were also looking to embrace change not only on the technology front, but they were looking for a partner who cared about their practice and provided top-notch service and support, additional differentiators that Fujifilm now consistently brings to its customers.

**L.L.: What have been the biggest challenges you’ve encountered in the United States compared with working in other countries?**

**T.F.:** Because of the required U.S. Food and Drug Administration (FDA) submissions and timelines for review, other countries and regions around the world have historically seen new technologies brought to market sooner. Our intent is to bring new technologies to the U.S. market as quickly as possible. But the given review timelines also allow us to hear from our international colleagues, and their customers, on the key differentiators and clinical utility of a new Fujifilm technology. This helps us better prepare to introduce that new technology to U.S. gastroenterology practices in ways that are meaningful to them.

**L.L.: What are lessons you’ve learned from working in the U.S. market?**

**T.F.:** Although we now have the most innovative portfolio of endoscopes in the industry, we also recognized it was equally important to scale our organization to offer the highest level of service and support. The service experience with Fujifilm starts at the initial product demonstration and continues through the life of each partnership. We find this approach and commitment to partnership—to being visible and present and supportive—is appreciated and welcomed by physicians, nurses, technicians, and staff alike.

**L.L.: Fujifilm Healthcare also has been highly successful at developing new scopes quite rapidly. Would you discuss the approach and how the company has been so nimble and responsive to clinician feedback?**

**Figure 7:**
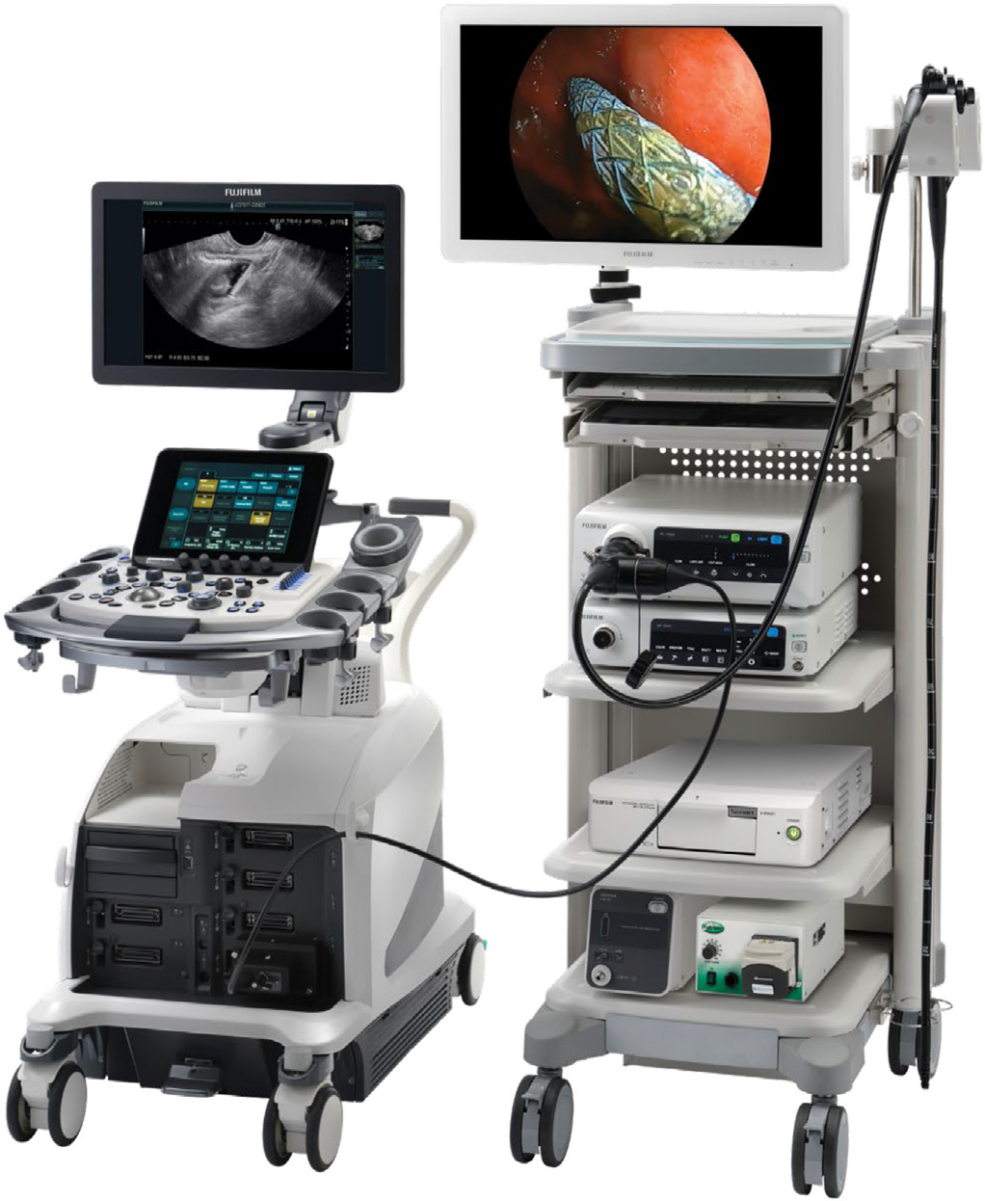
Fujifilm Arietta and EG-740UT echoendoscope.

**T.F.:** In our ongoing discussions with physicians, nurses, and technicians, it became apparent that there were gaps that needed to be addressed, and Fujifilm has been able to respond positively because we listen and take action. Although Fujifilm is a large and complex $20+ billion global company, the endoscopy division has proven to be nimble and able to pivot fairly quickly, operating with a startup mentality in many ways, with consistent support from the highest levels of the organization. Building on the success we’ve had in U.S. market delivering our latest state-of-the-art portfolio colonoscopes and gastroscopes, we’ve been able to deliver new innovations, for example, in EUS with the game-changing EG-740UT linear echoendoscope with novel forward-viewing capability (the objective lens is placed behind the elevator). Used in combination with the Fujifilm Arietta 850, this duo empowers endoscopists with the ability to obtain unmatched visualization and capability to perform complex lumen-apposing metal stent placement and other procedures (Fig. 7).

**Figure 8:**
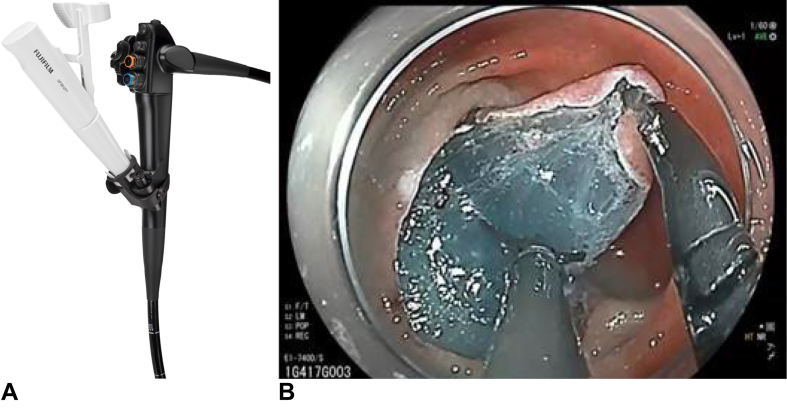
**A,** Fujifilm dual-channel scope with TRACMOTION. **B,** TRACMOTON during endoscopic submucosal dissection.

Fujifilm also realized that as GI tract diseases, conditions, and cancer treatments become more prevalent in the United States, this led to increasing performance of endoscopic submucosal dissection (ESD), peroral endoscopic myotomy, and bariatric suturing procedures. We introduced a new dual-channel endoscope that is FDA approved for both upper and lower GI applications, often used in performing these procedures. Additionally, we realized endoscopes were not all we want to deliver to help address these gastroenterology challenges. Fujifilm has added to its single-use device portfolio by introducing a novel tool to aid ESD, called TRACMOTION, a single-operator, 360-degree, rotatable grasper that is helping to simplify the lesion excision process and allowing for greater control and maneuverability during the procedure (Fig. 8). This device has the potential to help expand the use of ESD for colorectal cancer treatment in the United States.

Additionally, Fujifilm seeks to drive innovation through enhanced visualization that aids procedural performance for bariatric and thoracic applications. In late 2021, Fujifilm received the FDA’s Breakthrough device designation with ELUXEO Vision: a technology that allows for the visualization of tissue oxygenation without the use of indocyanine green dye, in real time, with both rigid and flexible endoscopes.

**L.L.: Is there such a thing as growing too rapidly? Has that been an issue at all, and, if so, how have you and your team mitigated against this?**

**T.F.:** Although we’ve seen significant growth in the endoscopy space, we’ve made a concerted effort in our approach to be smart in our growth strategy. We have forward built our infrastructure with the goal of exceeding our customers’ expectations at every point in our relationship. We’ve realized that physicians and their teams truly appreciate the level of support Fujifilm offers and the expertise and know-how we deliver in training, serving, and partnering with them.

**L.L.: Service is such an important aspect of your business. How have you approached ensuring optimal, timely service of your equipment throughout the United States and around the world?**

**T.F.:** Our commitment to innovation, although crucial and a key differentiator for Fujifilm, is only 1 leg of the stool, so to speak. Service and support are the other legs, and we’ve developed a business culture that is committed to exceeding customer expectations as it relates to all 3. As we continue to experience significant growth in market share, we have simultaneously invested internally to ensure we can support our customer needs, not only with their installations, repairs, and inventory management, but also with training and ongoing support.

**L.L.: I believe this is the 90th anniversary of Fujifilm. Looking forward, what does the future hold for Fujifilm Healthcare in the gastroenterology arena both within and outside the United States?**

**Figure 9:**
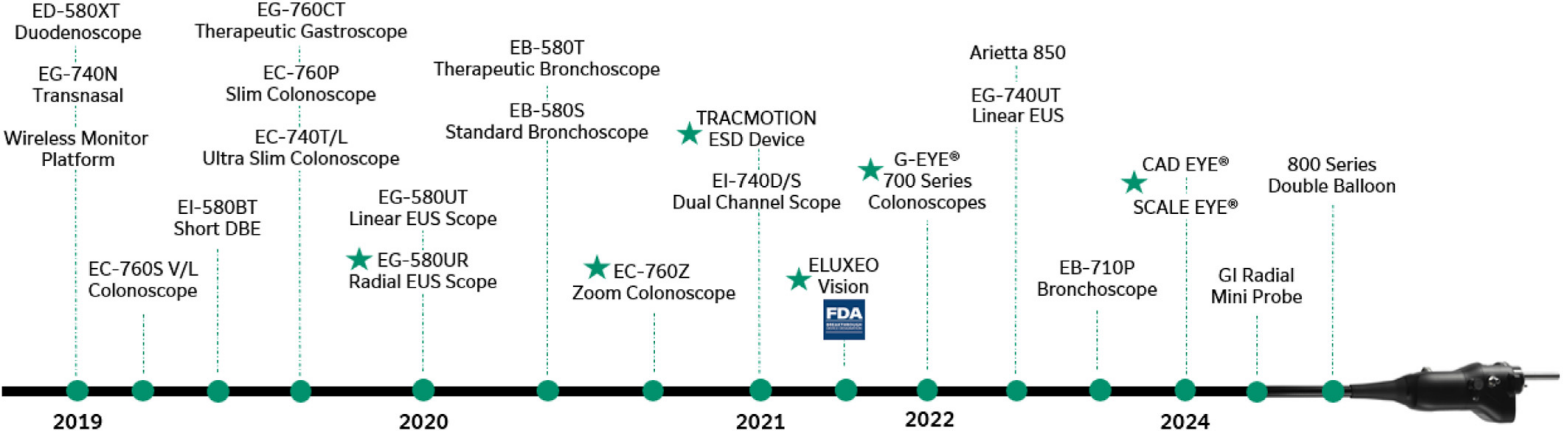
Fujifilm Endoscopy timeline of technology introductions to the U.S. market, 2019 to 2024. More than 20 new market products have been introduced in 5 years. *ESD*, Endoscopic submucosal dissection.

**Figure 10:**
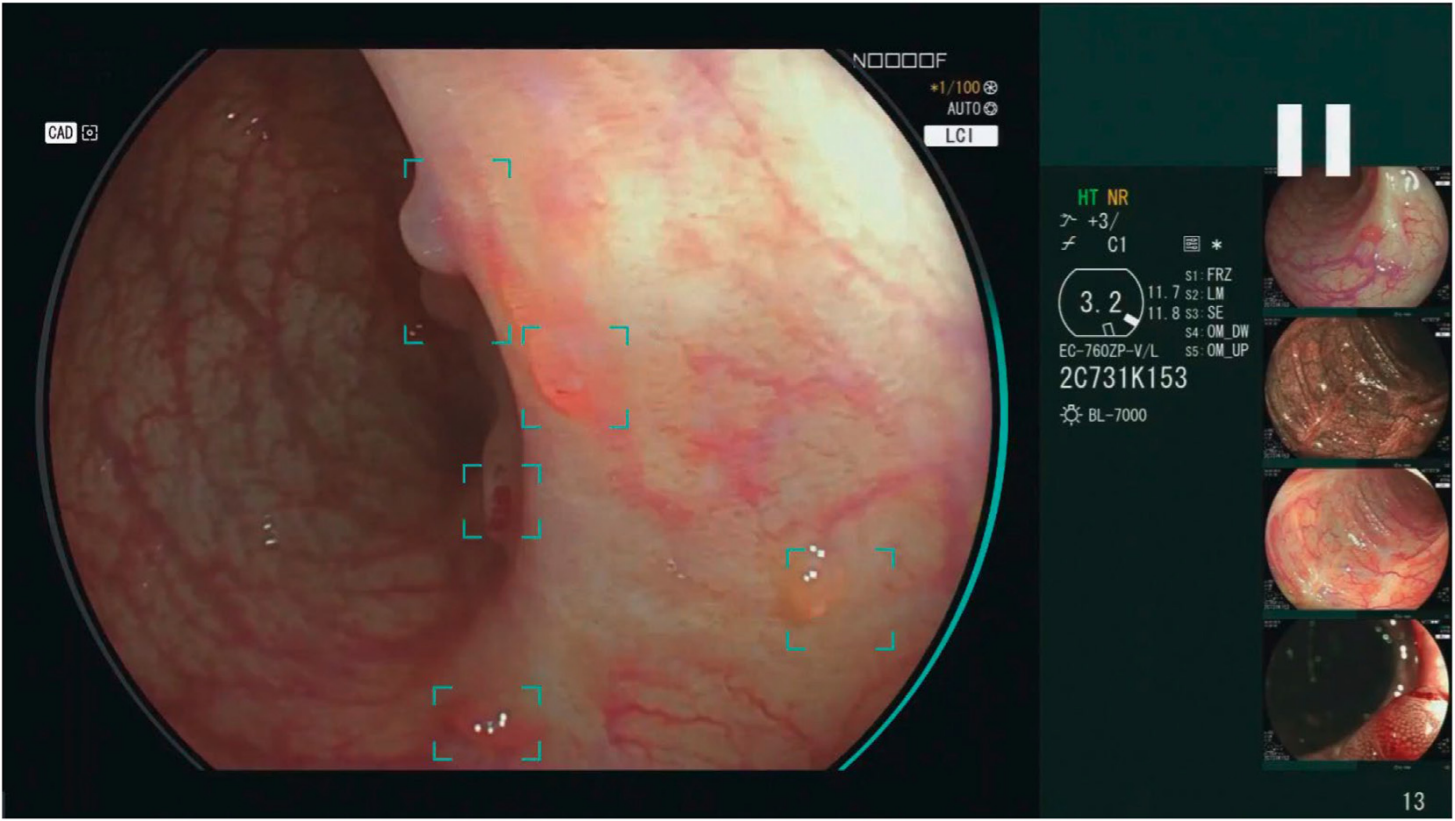
Fujifilm CAD EYE for colon polyp detection.

**T.F.:** At Fujifilm, we’re proud of our heritage of innovation and our overall commitment to the endoscopy space. Fujifilm continues to build on its mantra of “Value from Innovation.” In addition to the 20+ gastroenterology technologies introduced to the U.S. market since 2018 (Fig. 9), we are aggressively investing in future technologies that will better enable physicians to treat their patients. This includes conventional endoscopic technologies, but also includes leveraging artificial intelligence to aid physicians from a clinical and operational standpoint. Some of that artificial intelligence–enabled technology is already coming to market this year in the form of CAD EYE to support real-time detection of colonic mucosal lesions and SCALE EYE for more accurate in vivo polyp measurements (Fig. 10).

**L.L.: Fujifilm itself is a huge company with many different components and interests, even in cosmetics. Would you briefly touch on some of the various parts of Fujifilm outside of medical? Also, how does Fujifilm Healthcare fit into such a large company, and what are the pros and cons of being part of such a huge corporation with various interests?**

**T.F.:** Fujifilm was established in 1934 as an imaging company delivering photographic film and cameras, providing consumer and commercial products and services. As digital technologies took hold, Fujifilm smartly transformed itself to an organization that, today, comprises 279 companies employing more than 72,000 people across healthcare, electronic, imaging, and business innovation sectors, with total revenues exceeding 20+ billion U.S. dollars, and with a focus and commitment to innovation and sustainability.

Outside of our healthcare portfolio, we have several business units that continue to innovate in areas like imaging, document management, and, yes, cosmetics. Although very different from the healthcare segment, there are transferable technologies and methodologies that can add value across the entire Fujifilm portfolio, including health care.

Fujifilm’s healthcare portfolio focuses on delivering value from innovation with products that focus on prevention, diagnosis, and treatment. Beyond medical devices, diagnostic imaging, and radiologic software, we are contributing to the health of people around the world by responding to unmet medical needs, early detection of diseases, and support for the development and manufacture of innovative vaccines and pharmaceuticals.

Innovation can come from many different areas. The culture at Fujifilm, regardless of which business unit, allows for innovations to flourish and evolve from a seedling of an idea to a technology that positively impacts patient care.

## Editor’s Closing Remarks

With the endoscope still being the central component for what gastroenterologists do, ongoing innovation is critical to providing more effective and safe care to our patients. Adopting a startup mentality, listening critically and carefully to its consumers, providing outstanding service, and drawing on technology from outside of health care appear to have been key components to the success of Fujifilm Medical in entering the U.S. market. Ongoing partnerships with all our partner endoscope and device companies are critical to drive innovation with the ultimate goal of improving access and delivery of health care to all our patients.

## Disclosure

The following author disclosed financial relationships: L. S. Lee: Consultant for Boston Scientific, Fujifilm Medical, and Fractyl. The other author disclosed no financial relationships.
